# Case report: intra-tendinous ganglion of the anterior cruciate ligament in a young footballer

**DOI:** 10.1186/1749-799X-1-11

**Published:** 2006-11-02

**Authors:** Christer Rolf, Thomas P Watson

**Affiliations:** 1 From the Sheffield Centre of Sports Medicine, Division of Clinical Sciences South, University Of Sheffield, UK

## Abstract

A 20-year-old male medical student and keen rugby player presented with a 12-month history of progressively worsening right knee pain and stiffness with no history of trauma. Clinical examination revealed effusion and posterior knee pain exacerbated by end range movement and an extension lag of 15 degrees. Physiotherapy to improve the range of motion proved unsuccessful. Magnetic resonance imaging showed that the ACL was grossly thickened and displaced by material reported as mucoid in nature. There were also areas of focally high signal in relation to its tibial attachment and intra osseous small cysts. Arthroscopic examination revealed a ganglion related to the tibial attachment of the ACL and gross thickening and discoloration of the ACL. Biopsies were taken showing foci of mucoid degeneration in the ACL. A large intra-ACL mass of brownish coloured tissue was excised arthroscopically. Already at 2 weeks follow up the patient had greatly improved range of movement and was pain free. However, upon returning to rugby, joint instability was noticed and a tear of the ACL was confirmed.

This rare clinical condition can be diagnosed with MRI and arthroscopic debridement effectively relieves symptoms. This case report illustrates that augmentation or reconstruction may end up being the definitive treatment for athletes. It may also offer some support to the argument that mucoid degeneration and ganglion cyst formation share a similar pathogenesis to intra-osseous cyst formation.

## Background

A ganglion is a cystic lesion containing mucin-rich fluid associated with a joint or tendon sheath [1]. Ganglia of the anterior cruciate ligament (ACL) are uncommon. The prevalence of ganglia associated with the ACL is reported to be 0.12–0.44% on MRI [2,3]. Mucoid degeneration is characterized by an increase in the mucoid ground substance in the connective tissue containing glycoprotein and mucoprotein [4]. The prevalence of mucoid degeneration of the ACL has been reported to be 0.43% [5].

Bergin et al. reported on the co-existence of mucoid degeneration and ganglia of the ACL. From 4221 knee MRI examinations they found 26 patients (0.62%) who had both mucoid degeneration and ganglia of the ACL. Although they can coexist, the existence of a relationship between the two is an area of debate [5-8]. The aetiology of both ganglion cysts and mucoid degeneration is unknown [9-11]. Further relationships with intra-osseous bone formation are not explained in the literature. ACL ganglia and mucoid degeneration can cause pain and decreased range of movement in the knee [12,3-15]. A limited number of cases have been described in the literature.

In this report we describe a case of intra ACL mucoid degeneration and ganglion cyst combined with intra-osseous cysts in a 20-year-old medical student and keen rugby player who had to stop playing because of gradually increasing dysfunction of his knee. The clinical findings, MRI, histology and arthroscopic findings and outcome are described.

## Case presentation

A 20-year-old male medical student presented with a 12-month history of right knee pain of insidious onset. He had been a regular amateur rugby union player for 12 years. No episodes of trauma and no previous knee problems were reported. The problem began as stiffness and pain and had worsened in the last 3 months to the extent that he had to stop playing. The patient described the pain as an intermittent deep ache. The pain was exacerbated by end of range flexion and extension.

Physical examination revealed right-sided posterior knee pain exacerbated by end range flexion and extension and mild effusion. Active range of movement was -15°–95° and passive range of movement -10°–100° (Fig 5 and 7). Atrophy of the right quadriceps was present. There was no joint-line tenderness on palpation. Anterior and posterior drawer and Lachmann tests were negative whilst compression rotation test was positive.

**Figure 1:**
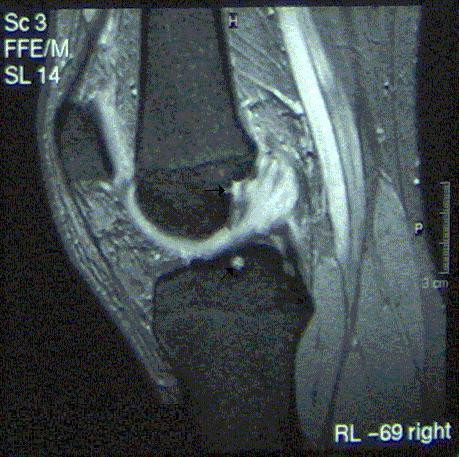
A sagittal T2-weighted MRI showing thickening and pathological appearance of the ACL.

Physiotherapy was initially undertaken in order to improve range of movement. At review no improvement at all was seen and compression rotation test was still positive. MRI scans showed an abnormal anterior cruciate ligament (ACL). Its fibres were intact but displaced by material containing high signal on the fat sat sequence, reported as appearing mucoid in nature. There were also areas of focally high signal in relation to its tibial attachment. These areas had the appearance of tiny ganglia. The report presumed that these ganglia extended into and thickened the ACL. The findings were in keeping with mucinous degeneration. There was evidence of intra-osseous cyst formation as shown by high signal at the attachment sites of the ligament (Fig. 1 – arrows). X-ray was unremarkable. Based on these findings an arthroscopy with biopsies was undertaken.

Arthroscopy revealed several small ganglia in the ACL around the tibial insertion. Both menisci were intact and cartilage was normal. The posterior cruciate ligament (PCL) was normal. The histology report from biopsies described ligament with multilocular foci of mucoid degeneration. There was no evidence of neoplasia.

**Figure 2:**
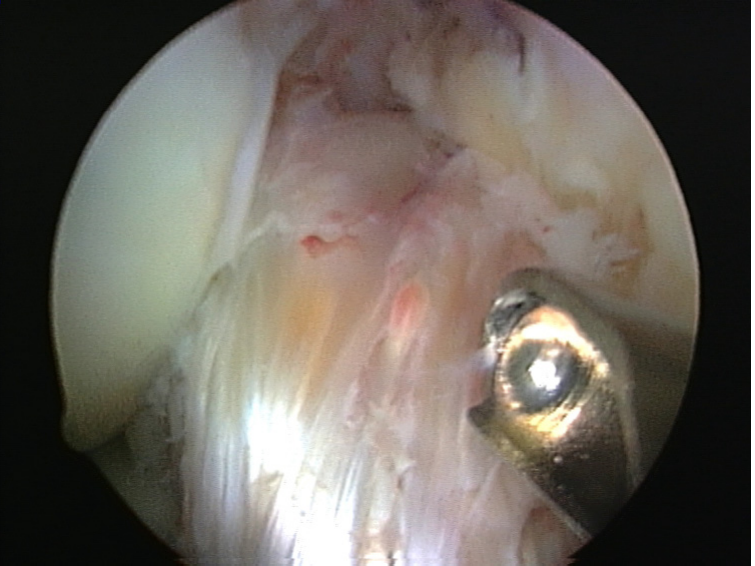
Abnormal tissue displacing the ACL anteriorly out of the notch.

**Figure 3:**
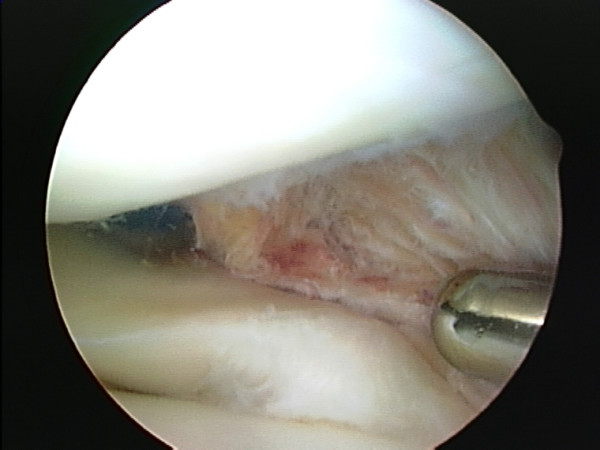
Posterior abnormal tissue close to the posterior lateral meniscus horn displacing the ACL.

**Figure 4:**
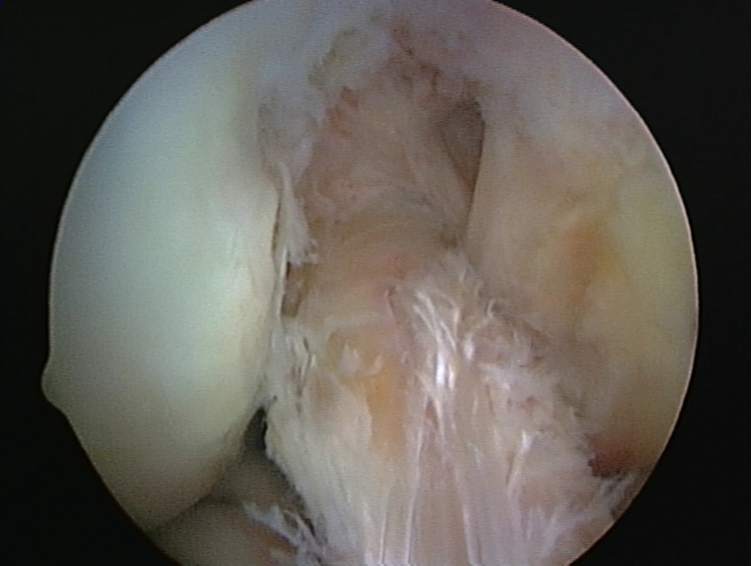
Improved position of ACL following excision of the diseased tissue.

As much as possible of the macroscopically abnormal tissue was excised arthroscopically (Fig 2, 3, 4). Examination under anaesthesia showed a range of movement of -15°–110° pre-operatively and -5 – 140 degrees post surgery.

**Figure 5:**
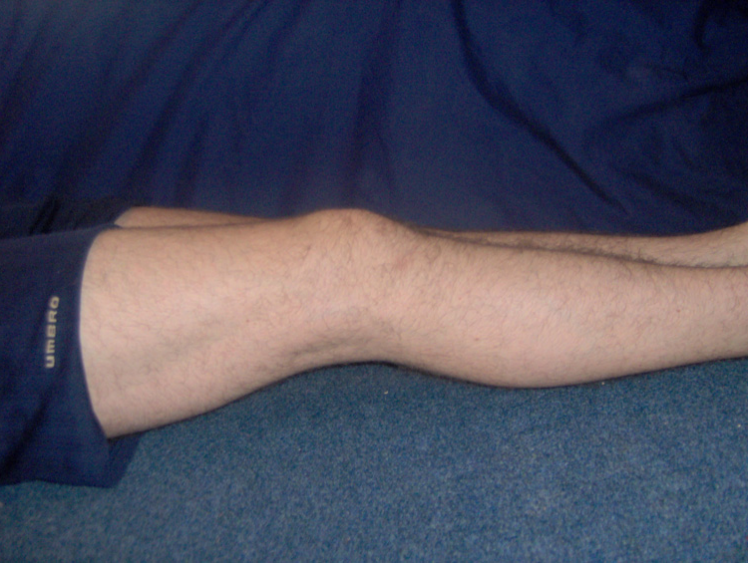
Reduced extension of the right leg prior to surgery.

**Figure 6:**
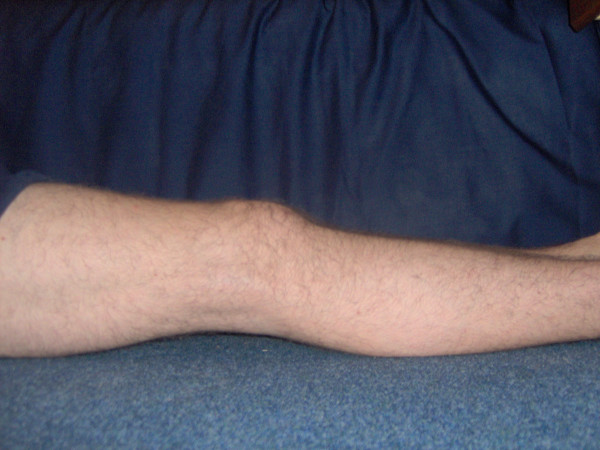
Improved extension 2 weeks post-operatively.

**Figure 7:**
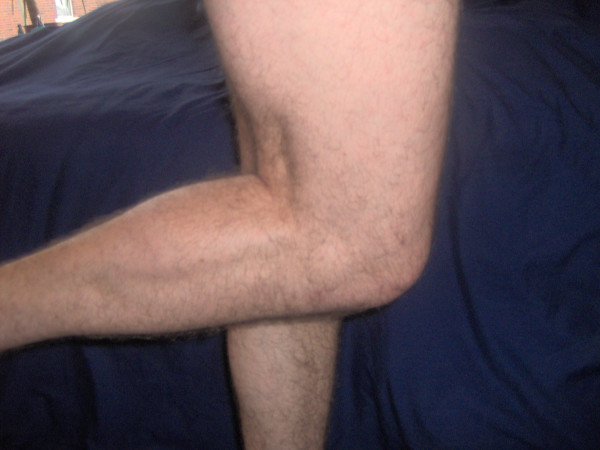
Reduced flexion of the right leg prior to surgery.

**Figure 8:**
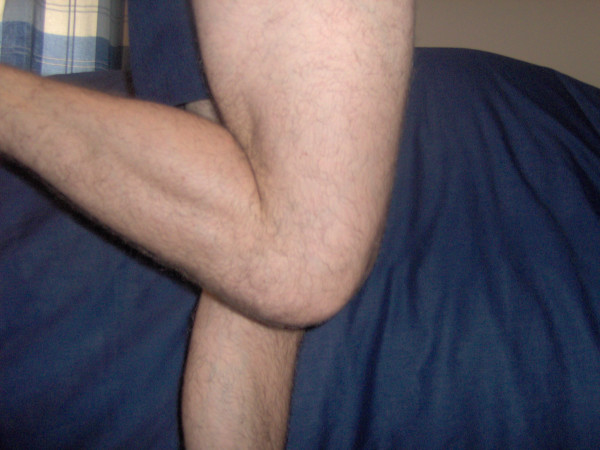
Improved flexion 2 weeks post-operatively.

Lachmann's test and anterior drawer tests were negative after extensive debridement. Post-operatively the range of movement was improved to 5°–140+° (Fig 5, 6, 7, 8). Following surgery the patient was referred for early mobilisation and physiotherapy. At 6 week follow up range of movement was increased to full passive flexion and full passive extension.

**Figure 9:**
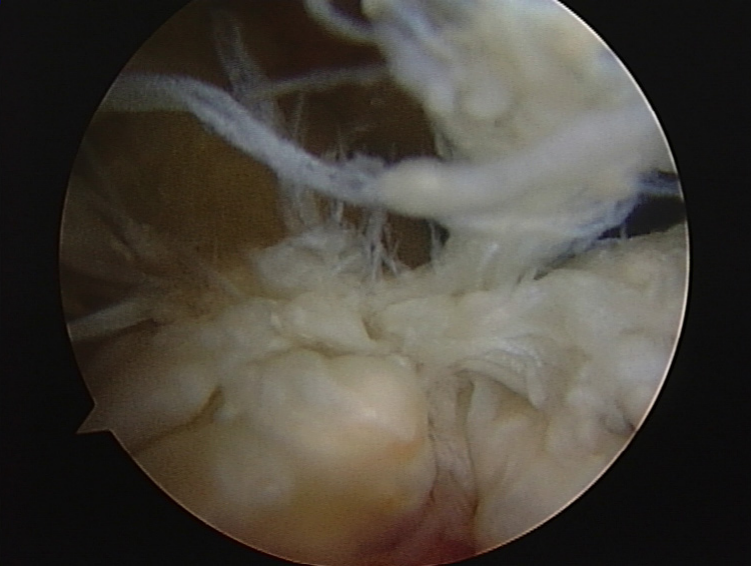
Torn ACL prior to reconstructive surgery.

The patient remained pain free and returned to rugby 14 weeks after operation. No major trauma occurred during this time. However, instability was described following several games and positive anterior drawer and Lachmann's tests were present on examination. Arthroscopy confirmed the presence of an ACL tear (Fig 9) and also showed cartilage and meniscal damage. The patient later had a patellar tendon graft performed.

## Discussion

For intra-tendinous ganglia of the ACL, MRI identified the lesion site,  although it was not entirely diagnostic. Arthroscopy and biopsy was necessary to rule out an early neoplastic process. Debridement of the abnormal mucoid tissue relieved symptoms effectively, which has been described previously in the literature [16,3,7,13,19]. However, we have not found as thorough documentation of clinical findings, MRI, histology, arthroscopy findings and outcomes as in the present case. Gradually decreased range of movement and stiffness of the knee joint in a young athlete without preceding trauma should therefore lead to this suspicion and an MRI and arthroscopy should be undertaken [3,11-13,20].

In these cases there is usually no preceding major trauma [8,7,10,12] or instability of the joint [5,16,17,12,13]. Common MRI findings are high signal on T2-weighted MRI images thickening the ACL with a 'celery-stalk' appearance [16,11,6,17,12,21], erosion of cortical bone [22,11,10] and intraosseous cyst formation [5,10]. Arthroscopically ligament fibres are interspersed with a yellow-brown substance and the ACL displaces anteriorly and posteriorly [3,11,12,21]. All of these features were seen in this case. Mucoid degeneration and ganglia of the anterior cruciate ligament are uncommon [7,9,13,3]. Further more so is their coexistence. Bergin et al. reported the prevalence of this to be 0.62% on MRI [5].

The aetiology of ganglion cysts and mucoid degeneration is unclear [9,11,10]. One theory is that mucoid degeneration leads to ganglia formation [7]. This relationship is commonly theorised in the literature but its existence is unproven. Bergin et al. reported that ACL ganglia and mucoid degeneration commonly coexist and gave some evidence to suggest these two entities may share a similar pathogenesis [5]. Another theory suggests that herniation of synovial tissue through a defect in the tendon sheath causes ganglia formation [15]. A third describes displacement of synovial tissue during embryogenesis [3]. The relationship to trauma is unknown. One theory involves the cellular response to trauma that liberates a mucin substance, hyaluronic acid. This is interspersed with the fibres of the ligament, causing its fusiform dilatation. With joint and tissue motion, the mucin substance dissects the ligament fibers and may be found at the ligament attachments or in the intercondylar notch of the knee [9]. Many cases in the literature describe ganglia formation in the absence of trauma. However, excessive training or repetitive minor trauma such as rugby tackles could well be a triggering factor [8,7,10,12]. Although repetitive trauma from rugby may be a contributing factor, the aetiology of the current case is not known and there are no known hereditary factors in the history.

There are no reported cases of ACL rupture following pathogenesis of this type. The literature shows that arthroscopic debridement of the abnormal tissue effectively relieves symptoms [16,3,6,13,19]. However, this inevitably results in a thinned ACL, which could compromise joint stability. Cases in the literature report no instability in day-to-day activities following debridement [16,11,17,12]. However, none of these patients played sport. Reporting on five cases, Narvekar et al concluded that because none of the patients participated in any type of sporting activity, the thinned ACL mass probably sufficed to provide the requisite stability for day-to-day activities [17]. Nishimori et al concluded that if their patients had participated in any type of sport, they might have had to consider augmentation or reconstruction of the ACL after resection of the lesion [12].

Only one previous case of an athlete is reported; Fealy et al describe a successful return to sport following arthroscopic debridement of the ACL of a volleyball player [16].

This rare diagnosis and treatment option should be considered when a young athlete presents with reduced ROM of the knee without preceding trauma.

Arthroscopic debridement of the abnormal tissue effectively relieves symptoms.

Augmentation or reconstruction of the ACL may end up being the definitive treatment if the patient returns to a sport demanding high levels of stability.

This report may also offer some support to the argument that mucoid degeneration and ganglion in the ACL and intraosseous cyst formation share a similar pathogenesis.

## Abbreviations

ACL – Anterior cruciate ligament

PCL – Posterior cruciate ligament

ROM – Range of movement

## Competing interests

The author(s) declare that they have no competing interests.

## Authors' contributions

CR conceived of the study, participated in its design and coordination and helped to draft the manuscript. CR revised the article for intellectual content details. TW conducted the literature review and carried out the review of the patient's medical record in order to collect all the available information. TW helped draft the manuscript. Both authors read and approved the final manuscript.
